# Functional Abdominal Pain Syndrome in Morbidly Obese Patients Following Laparoscopic Gastric Bypass Surgery

**DOI:** 10.5812/atr.13110

**Published:** 2014-03-20

**Authors:** Mohammad Eidy, Abdolreza Pazouki, Fahimeh Raygan, Yazdan Ariyazand, Mohadeseh Pishgahroudsari, Fatemeh Jesmi

**Affiliations:** 1 Trauma Research Center, Kashan University of Medical Sciences, Kashan, IR Iran; 2 Minimally Invasive Surgery Research Centre, Iran University of Medical Sciences, Tehran, IR Iran

**Keywords:** Surgical Procedures, Minimally Invasive, Abdominal Pain, LGBP Protein, Pacifastacus Leniusculus

## Abstract

**Background:**

Roux-en-Y gastric bypass surgery (RYGBP) is one of the most common bariatric surgeries, which is being performed using various techniques like gastrojejunostomy by hand swen, linear or circular stapler. Abdominal pain is a common complaint following laparoscopic gastric bypass procedure (LGBP), which has different aetiologies, such as overeating, adhesion, internal herniation, bile reflux and many more. In this study LGBP was performed in an ante-colic ante-gastric pattern in a double loop manner and the prevalence and distribution of pain in morbidly obese patients undergoing LGBP was assessed.

**Objectives:**

The aim of this study was to analyze the distribution and frequency of post LGBP pain in morbidly obese patients.

**Patients and Methods:**

This study was performed on 190 morbidly obese patients referred to Hazrat Rasoul Hospital in Tehran. After LGBP, pain was measured in the following intervals: 24 hours, one week and one month after the operation. Before the operation onset, 2 mg Keflin and 5000 IU subcutaneous heparin were administered as prophylaxis. LGBP was performed using five ports including: one 11 mm port was placed 15-20 cm far from the xiphoid, one 12-mm port in mid-clavicular line at the level of camera port, one 5-mm port in subcostal area in ante-axillary region in the left, another 5-mm port in the right mid-clavicular area and a 5-mm port in sub-xyphoid. All operations were done by the same team. Staple was used for all anastomoses and hand sewn technique to close the staple insertion site. The mesenteric defect was left open and no effort was made to repair it.

**Results:**

The results of this study showed that 99.94 % of the patients had complains of pain in the first 24 hours of post operation, about 60% after one week and 29.5 % still had pain after one month. In addition, left upper quadrant (LUQ) was found to be the most prevalent site for the pain in 53.7% of the patients in the first 24 hours, 59.6% after one week and 16.8% after one month (except for obscure pain) with a significance of < 0.05.

**Conclusions:**

In this study, the authors analyzed the location and disturbance level of pain after LGBP, which could serve as a cornerstone for further researches. The authors suggest that long-term follow-up (for more than a year after operation) should be considered in future studies and also the relationship between the drainage site and pain should be investigated.

## 1. Background

There are increasing numbers of requests for gastric bypass surgery due to the global obesity growth. The annual bariatric surgeries in USA are about 20000 (1). Roux-en-y gastric bypass surgery (RYGBP) is one of the most common bariatric surgeries, which seems to be the gold standard operation for morbid obesity. Although it is considered as a minimally invasive surgery, most of the patients complain of abdominal pain, after laparoscopic gastric bypass (LGBP) (2, 3). In one study it has been reported that 45% of patients admitted to the emergency department complained of abdominal pain. About 54.1% of these patients were referred again for the second time and 22.6% were admitted for the third time, all due to abdominal pain (2).There are many reasons for pain after gastric bypass surgeries, several of which could be related to the behavioral eating patterns, such as rapid eating, (4), food intolerance, like rice, vegetables, fruits, pasta, gluten and lactose intolerance, some deficiencies like Folate and vitamin B12 deficiencies (5) and finally bacterial overgrowth (6). 

Obesity causes changes in bowel habit and these changes are more obvious after surgery. Most patients go through functional changes in the gastro intestinal tract after surgery, most common being constipated and lower abdominal pain (7, 8). There are similar changes in esophageal activity that cause retrosternal pain, most cases relieve in time (9). The gallbladder stone is believed to cause right upper quadrant (RUQ) pain (10, 11). Changing in Oddi sphincter function (12), the issue of ulcers in the gastric pouch (13, 14) and gastroesophageal reflux disease (GERD) (15, 16) have been known as the leading causes of pain. A rare cause of pain is anastomotic stenosis, which could happen in 5-10% of patients and mostly in the first three months, post operatively (17, 18). Trocar site hernia is reported in less than 1% of patients (19) and adhesion band in 12% of patients with the most common site being the jejenojejenostomy site (19). Incisional hernia was seen in 1-9% patients especially in the first 3 days of post-operation (17, 18). Some articles have reported intussusception in 1% of cases in months or years after surgery (19). Jejenostomy site stenosis was reported in 5% of the patients (20). Other rare causes could be omental torsion and infarction (21, 22) and bezoars (23, 24). For these reasons, many studies have suggested different remedies to relieve the abdominal pain, yet none has been proved to be sufficient.

To the best of our knowledge, no previous studies have been done to analyze the distribution and frequency of post LGBP pain in patients.

## 2. Objectives

The aim of this study was to analyze the distribution and frequency of post LGBP pain in morbidly obese patients.

## 3. Patients and Methods

In this study, 190 morbidly obese patients with BMI > 35 were selected. They all had concomitant diseases, such as hyper metabolic conditions, DM, HTN and HLP, pulmonary diseases like asthma, non-alcoholic steatosis, stress incontinency, venous stasis, hyper ventilation syndrome and GERD. All patients’ surgeries were in Hazrat Rasoul Hospital from May 2012 to June 2013. Classic RYGBP with a double technique was performed in an ante-colic anti-gastric pattern. Before the onset of the operation 2 mg Keflin and 5000 IU subcutaneous heparin were administered as prophylaxis. LGBP was performed using five ports including: one 11-mm port, 15-20 cm far from the xiphoid, one 12-mm port in mid-clavicular line at the level of camera port, one 5-mm port in subcostal area in ante-axillary region in the left and another 5-mm port in the right mid-clavicular area and a 5-mm port placed in sub-xiphoid. All operations were performed by the same team. Staple was used for all anastomoses and hand sewn technique to close the staple insertion site. The mesenteric defect was left open and no effort was made to repair it. At the end of operation leak test was performed for all cases. Penrose drains were placed for each patient. The day after the operation, gastrografin upper GI series was performed. The case was excluded from the study if the surgeon was encountered with any case of positive leak test or had to convert the operation into open surgery. A questionnaire was edited and filled for each patient in first 24 hours, one week, and one month after the surgery. The questionnaire contained pain scoring according to the visual analogue pain scale, the quality and quantity of pain, its distribution, association with the drained site and other port entry sites, and also how the patient could manage to reduce the pain. Pain scoring system in this study was based on VAS method with the scales 1 to 10. Scores of 1-3 were considered as mild, 4-6 were categorized as moderate and 7-10 scores were considered as high or severe pain. Finally all data were analyzed by SPSS software (version 11.5). The quantitative data were expressed as the mean standard deviation (SD), and the frequency was used for the qualitative data. For comparing the qualitative data, chi-square or Fisher’s exact test was used. A p-value less than 0.05 was considered as statistically significant.

This trial was registered at http://www.irct.ir. The registration number of this trial was IRCT201202198588N3.

## 4. Results

A total number of 190 patients underwent laparoscopic RYGBP. The mean age of patients was 37.93 (± 8.47; 24-52) years, 145 patients (76.3%) were female and 45 patients (23.7%) were male. The mean BMI of patients was 41.84 ± 6.17. At the first week, 188 (98.9%) of patients had abdominal pain, by the next week 114 (60%) and by 5th week, 56 (29.5%) had abdominal pain (Table 1). At week 0, the highest incidence of abdominal pain after laparoscopic RYGBP was reported by 101 patients (53.7%) in LUQ, 103 (54.8%) of patients had severe pain (P < 0.001). By the first week, the highest incidence of abdominal pain after surgery was in LUQ in 68 patients (59.6%), and severity of pain in all these cases was mild, 59 (51.8), (P < 0.001), and by week 4, the highest incidence of abdominal pain was in the LUQ in 32 patients (57.1%), and at this time, severity of pain in the most patients 27 (49.1%) was mild (P < 0.001) (Table 2). There was no correlation between age and severity of pain at any time (P value > 0.05 for week 0, week 1 and week 4). There was a significant correlation between BMI and severity of pain at week 4 following the surgery (P value < 0.0001, r = 0.490).

**Table 1.** The Prevalence and Abdominal Pain Severity Following Laparoscopic RYGBP ^a^

Severity of Pain, Time	No Pain	Mild	Moderate	Severe
**Week 0**	2 (1.1)	48 (25.3)	37 (19.5)	103 (54.2)
**Week 1**	76 (40.0)	59 (31.1)	55 (28.9)	0 (0.0)
**Week 4**	134 (70.5)	24 (12.6)	8 (4.2)	24 (12.6)

^a^ The data are expressed as No. (%).

**Table 2.** Pain Distribution and Abdominal Pain Severity Following Laparoscopic RYGBP ^a^

Pain Site	Mild	Moderate	Severe	Total
**Week 0 (24 hours after surgery)**				
Epigastric	8 (53.3)	7 (46.7)	0 (0.0)	15 (8.0)
LUQ	8 (7.9)	22 (21.8)	71 (70.3)	101 (53.7)
RUQ	3 (100)	0 (0.0)	0 (0.0)	3 (1.6)
Unspecific area	29 (42.0)	8 (11.6)	32 (46.4)	69 (36.7)
Total	48 (25.5)	37 (19.7)	103 (54.8)	188 (100)
**Week 1**				
Epigastric	8 (100)	0 (0.0)	0 (0.0)	8 (7.0)
LUQ	16 (23.5)	52 (76.5)	0 (0.0)	68 (59.6)
RUQ	0 (0.0)	3 (100)	0 (0.0)	3 (2.6)
Unspecific area	35 (100)	0 (0.0)	0 (0.0)	35 (30.7)
Total	59 (51.8)	55 (48.2)	0 (0.0)	114 (100)
**Week 4**				
Epigastric	16 (100)	0 (0.0)	0 (0.0)	16 (29.1)
LUQ	3 (9.7)	8 (25.8)	20 (64.5)	31 (56.4)
RUQ	8 (100)	0 (0.0)	0 (0.0)	8 (14.5)
Unspecific area	0 (0.0)	0 (0.0)	0 (0.0)	0 (0.0)
Total	27 (49.1)	8 (14.5)	20 (36.4)	55 (100)

^a^ The data are expressed as No. (%).

## 5. Discussion

There are different aetiologies for pain following visceral surgeries (25), although with minimally invasive techniques and as a result of minimally access incisions, there has been a great reduction (26, 27) but due to visceral dissection, resection and some peritoneal disruptions, these patients experience pain too. Visceral pain is different from somatic pain (28) in visceral forms, the pain is transmitted with the enteric nervous system and not by the central nervous system (29). Similar to other minimal invasive operations, the pain following gastric bypass surgery is a serious problem, especially the late pain and the aim of this study was to discuss the prevalence of this pain to find a solution for the problem.

Morbid obesity is one of the major problems in the world, as in the US more than 30% of the population have this problem (26) and the surgical intervention is the only effective treatment. Gastric bypass is the most common bariatric surgery performed on these patients. In US more than 100,000 gastric bypasses are performed annually (27). Chronic abdominal pain is reported in less than 61% of patients, which might have different aetiologies as explained, (27) but in less than 15% no aetiology can be found (29). Minyoung Cho and his colleagues categorized pain after gastric bypass surgery based on admission to emergency following the operation, but in this study, the authors have categorized pain based on its existence after surgery and in the pre-mentioned time intervals. Therefore 98.94% of patients were found to have pain in the first 24 hours of post-surgery, 60% at the end of the first week and 29.5% still had pain four weeks after the surgery. No relation was found between pain and BMI, age, or gender. In this study only existence and localization of pain was checked. The authors explained that there is a special way for distribution of pain based on the time passed after operation. As the result of this study shows the highest incidence of abdominal pain was in LUQ after 24 hours (53.7%), at the end of the first week (56.9%), and also at the end of the fourth week (16.8%) It could be concluded that in all the three time intervals pain was mostly evident in LUQ. There have been numerous surveys conducted to achieve the goal of reducing pain after gastric bypass surgeries. In one study intraperitoneal bupivacaine was used at the end of procedure to reduce narcotic intake and post-operative abdominal and shoulder pain (31). Although no statistical difference was found between intraperitoneal bupivacaine and narcotic usage, this method could be beneficial (30). In a study by Emmanuel and colleagues, 75 among 1500 patients underwent LGBP in an ante-colicante-gastric pattern, were selected. The selected population all had defect closure and abdominal pain after the surgery. CT scan was performed on all 75 patients and it was revealed that 40 of them had internal herniation and signs of obstruction, and the other 35 had no signs. The diagnostic laparoscopy was performed and 29 of those 35 patients had internal herniation of tight adhesion bands. Consequently, the authors of the mentioned paper concluded that by the means of diagnostic laparoscopy the reason for 92% of abdominal pain could be revealed unless the pain reliefs spontaneously (31). In the present study the focus was on existence and localization of pain rather than its cause. The authors believe that by localizing the pain it would be possible to accurately diagnose the reason of it and hopefully go for proper treatment. It would also give the surgeon a better approach to reach the main origin of pain. For example the results of this study showed that the most painful area after LGBP surgery would be LUQ in the first day, week and month post-operatively. All drains were extracted from left flank, as it is routinely done in most LGBPs. It is not known if there is any relation between abdominal pain and site of the drain extraction as it is in laparoscopic cholecystectomy (32). Pain in the first day and at the end of first week seems logical but the reason behind remained pain in LUQ site after a month is still unknown (to the author’s knowledge) and more research should be conducted to clarify this. The limitations of this study can be due to the study duration (one month) and no long-term follow-ups. The authors suggest that long-term follow-up (for more than a year after operation) should be considered in future studies and also the relationship between the drainage site and pain should be investigated.
